# Using Ellenberg-Pignatti values to estimate habitat preferences of wild food and medicinal plants: an example from northeastern Istria (Croatia)

**DOI:** 10.1186/s13002-017-0159-6

**Published:** 2017-06-02

**Authors:** Ivana Vitasović Kosić, Josip Juračak, Łukasz Łuczaj

**Affiliations:** 1 Department of Agricultural Botany, University of Zagreb Faculty of Agriculture, Svetošimunska 25, 10000 Zagreb, Croatia; 2 Department of Management and Rural Entrepreneurship, University of Zagreb Faculty of Agriculture, Svetošimunska 25, 10000 Zagreb, Croatia; 3 Department of Botany, Institute of Biotechnology, University of Rzeszów, Werynia 502, 36-100 Kolbuszowa, Poland

**Keywords:** Ethnoecology, Quantitative ethnobotany, Ellenberg indicator values, Wild edible plants, Medicinal plants, Ćićarija, Phytoindication, Ethnobotany, Ethnomedicine

## Abstract

**Background:**

The paper presents the first ethnobotanical application of Ellenberg indicator values, which are widely used in European plant ecology. The aim of the study was to find out if Ellenberg values (indicating habitat preferences) differ for wild food and medicinal plants used in north-eastern Istria (Croatia). We used Ellenberg-Pignatti values (the version of Ellenberg values used in this part of Europe).

**Methods:**

Fifty semi-structured interviews were carried out among local key informants, asking which wild food and medicinal plants they used.

**Results:**

The mean number of food and medicinal plants mentioned per interview was 30. Altogether, 121 species were recorded as food or medicine used or previously used in the study area. Thirty-one species are used exclusively as food or everyday drink, 50 species are used exclusively as medicine and 40 species are used for both food and medicine. There were no significant differences between Ellenberg values for food and medicinal plants, apart from the Nitrogen indicator value – the plants used exclusively as food had a significantly higher index than those used in medicine. This probably stems from the fact that plants with soft fleshy shoots are attractive as food and they are more likely to come from nitrogen-rich ruderal habitats.

**Conclusions:**

Food plants and medicinal plants are collected from a variety of habitats and no clear difference between the two categories of plants was detected, however further testing of Ellenberg values in ethnobotanical studies could be interesting.

## Background

Ellenberg values are indices given to each species in a flora to express the species’ environmental preferences [1, 2]. The system was first introduced by the prominent German phytosociologist Heinz Ellenberg (1913–1997), and applied to the vegetation of central Europe. It consists of the following indices: Light, Temperature, Continentality, Soil Moisture, Reaction, Nitrogen and Salinity. Later it was modified to incorporate local differences in species’ environmental preferences, e.g. in Switzerland, Poland, British Isles and Italy [3–6]. Ellenberg-Pignatti values from Italy are used in the Mediterranean part of Croatia, as the climate and vegetation zones of Croatia bear many similarities to those of the adjacent Italy.

The values are usually given on a ten grade scale (0–9), apart from Light, Temperature and Soil Moisture, expressed on a 0–12 scale and salinity on a 0–3 scale. They are based on the field experiences of ecologists. Although looking at single species values does not have much practical use, comparison of average values for different sites and habitats can be used for phytoindication and characterizing environmental conditions at a given site [7, 8]. Some reductionist-oriented ecologists criticise the values for being based on biased choices and impressions and for mixing the absolute requirements of species with their ecological niches, which are the results of competition [9, 10]. Being aware of the constraints in using these values in our article, we aimed at applying them to ethnobotanical data.

The data matrix in an ethnobotanical study has many similarities with a phytosociological study. In the latter we obtain a data sheet composed of a species x releve matrix, whereas in the former we usually have a similar species x informant matrix which is later analysed. In both cases the same indices and tools may be used to describe the data: species frequency, diversity indices, ordination methods etc. Increasing quantification of ethnobotanical studies has been continuously advocated by some ethnobotanists, particularly by Ulysses de Albuquerque and his colleagues [11, 12]. This would strengthen the discipline and provide rigorous testing methods. Species frequency and diversity indices are probably used in at least half of ethnobotanical papers. However, other numerical methods and tools used in ecology, such as ordination techniques (see e.g. [13] from the similar study area), are used less frequently.

Traditional knowledge usually extends to different habitats surrounding human settlements. However, it has been noticed that it is not evenly distributed. For example ruderal “weedy” species tend to be over-utilized compared to the species of primary forest habitats [14, 15], although the opposite can be true in some cultures as well [16]. Writing this paper we wanted to explore the use of Ellenberg values to establish whether there are differences between the environmental preferences of wild medicinal and food plants. We assumed that some Ellenberg values would be different for food and medicinal plants, as medicinal plants are often rich in essential oils and alkaloids, so they would tend to grow in dry pastures and grasslands, while food plants should be gathered from more nutrient-rich and mesic habitats. An additional aim was to document the use of plants from the whole food-medicine spectrum in the area.

For our case study we used the area of Ćićarija in the north-eastern part of the Istria peninsula. Only two ethnobotanical studies dealing with plant remedies had previously been carried out there, covering several villages in the core part of Ćićarija [13, 17]. One of these studies concerned Croatians [13], another – Istro-Romanians [17]. In this paper we extended the research topic to food plants and included more villages from the area at the base of Ćićarija. The study was made easier by the fact that the first authors had carried out long term phytosociological research in the same area between 2003 and 2015 [18–21] and acquired a good level of knowledge about the local community and available key informants over the years. Our study was restricted to inhabitants of Croatian nationality.

### Study area

**Fig. 1:**
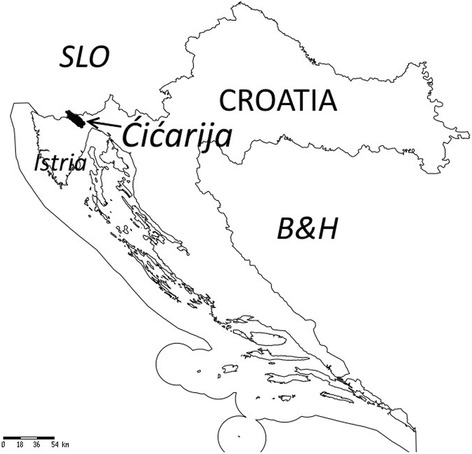
Location of Ćićarija in Croatia

**Fig. 2:**
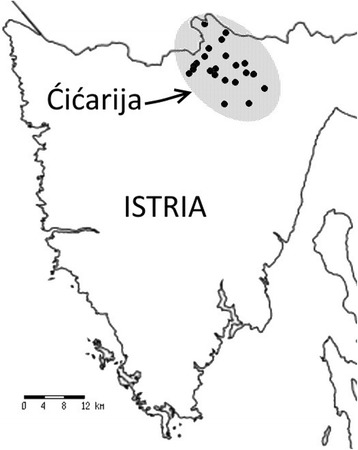
Location of the Ćićarija villages in the study area

**Fig. 3:**
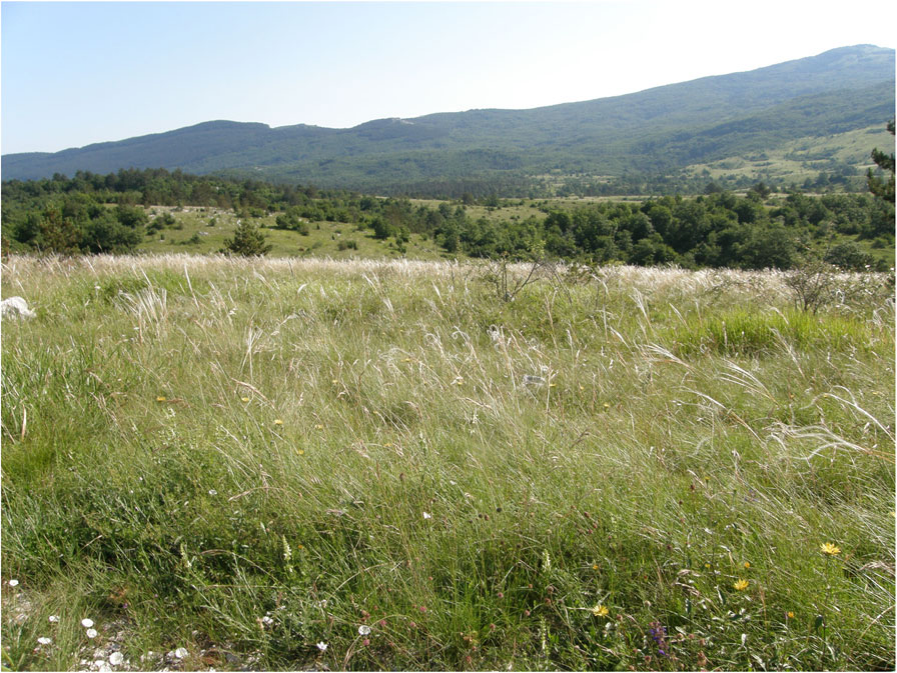
The landscape of abandoned pastures in the study area

**Fig. 4:**
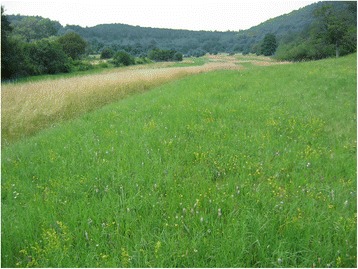
Hay meadows and forests in the study area

**Fig. 5:**
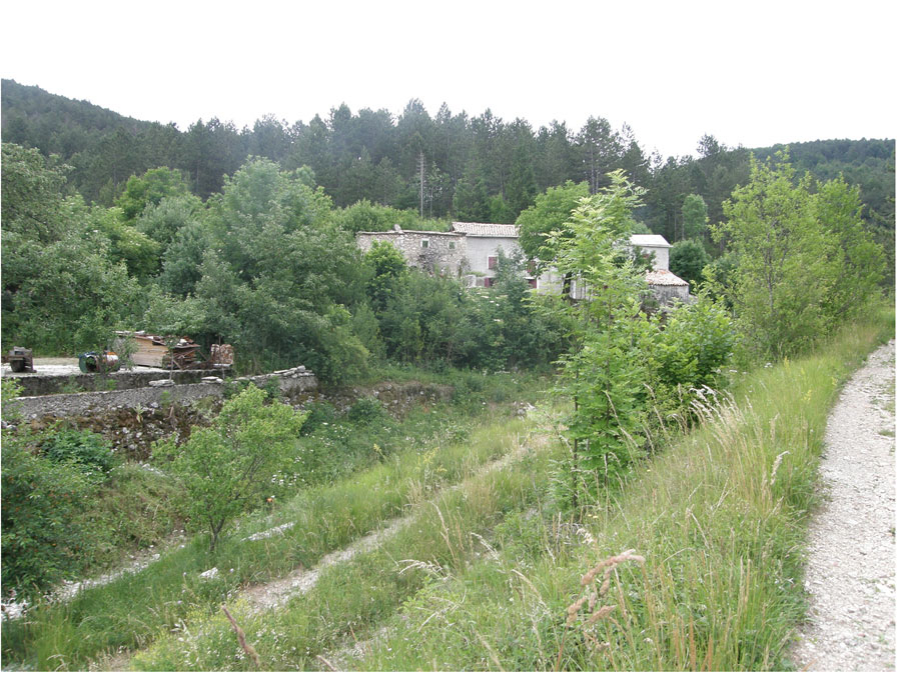
A view of a settlement in the study area

The study area (about 1000 ha) is part of the North Adriatic Karst and is located in Croatia at the north of the Istrian Peninsula, on the Ćićarija (Ital. Cicceria, Monti della Vena) mountainous plateau (45° 29′ 56″–45° 30′ 00″N, 13° 59′ 54″–14° 00′ 29″E), ranging 250–900 m a.s.l (Figs. 1 and 2). The name Ćićarija is derived from the South Slavic term Ćić, which refers to Istrians living north and north-east of the Učka mountain, originally referring to the Vlachs and Istro-Romanians of the area [22]. The area belongs to the Special Protection Area (SPA) of the Natura 2000 network (92/43/EEC Directive) as an important site for habitat 62A0 (Eastern sub-mediterranean dry grasslands *Scorzoneratalia villosae* H-ić 1975) and bird species conservation. The climate is transitional between Mediterranean and continental pre-Alpine, with cool, rainy winters and long, dry summers [23]. The mean annual temperature is 12.6 °C, the coldest in February (0–2 °C) and warmest in July or August (18–22 °C). Precipitation is about 1500 mm/year, most of which falls in autumn; a less pronounced secondary peak occurs as spring turns to summer. From a bioclimatic viewpoint, the study area belongs to the sub-Mediterranean belt [24] and the epi-Mediterranean mountain zone. The territory is characterised by karstic phenomena (dolines, caves, etc.); the bedrock consists of limestone; soils are generally brown, shallow and clast-rich. Pastures are for the most part under-grazed because of a low density of grazers (sheep) or abandoned (Fig. 3); meadows are irregularly mown, abandoned or, in some cases, derive from seeded forage meadows that have been abandoned [18]. In the past (pre-World War II) most of the Ćićarija territory was karst and grassland. The pastoral landscape is characterized by pastures and meadows, belonging to the *Scorzoneretalia villosae* order (*Festuco-Brometea* class) and *Arrhenatheretalia elatioris* order (*Molinio-Arrhenatheretea* class; Fig. 4), respectively [18–21].The potential natural vegetation of most of the area is composed of sub-Mediterranean forests of *Quercetalia pubescentis* Br.-Bl. (1931) 1932 order and, at higher elevations, *Fagetalia sylvaticea* Pawl. 1928 [21]. A complex mosaic of ruderal vegetation occurs in the villages (Fig.5).

For many centuries Istria was at the cross-roads of cultural exchange and trade between the Austrian Empire and the Republic of Venice. Ćićarija, especially, was an area of frequent migrations due to wars and other disastrous events like plague epidemics. Therefore, even today it is considered as an area of multicultural interactions between peoples that have settled here throughout history (Croatians, Istro-Romanians, Slovenians, Italians and Austrians), and many people are multilingual. These historical events have influenced the creation of traditions and the mentality of people on this mountain.

In this region sheep grazing and the herding way of life has been present for centuries. At the time of agrarian overpopulation (the 19th and the beginning of the twentieth century) large areas of the karst area in Ćićarija were turned into barren, rocky grasslands, almost desert regions which were later afforested (mostly with *Pinus nigra*). After World War II, traditional ways of farming have been abandoned and there is a movement towards the farmyard way of livestock breeding [26], as well as the termination of transhumance from the southern parts of Istria, or even from distant regions (Bosnia and Herzegovina and Macedonia). Back in 1869 there were 160,000 sheep in Istria, whereas in 2003 there were only 12, 000 [25]. Nowadays in the area described there are about 200 sheep (I.J., information from local farmers).

Therefore, today in the area of Ćićarija negative trends such as the abandonment of land, secondary succession, large-scale fires, reducing the number of population in distant areas, the disappearance of plant and animal species (loss of biodiversity) are present [26]. With the entry into force of the European “green” agricultural policy, agri-environmental measures have been taken to prevent further succession, to preserve the cultural landscape and biodiversity and to increase population density. Today the area of Ćićarija is populated by a predominantly elderly population, the younger people are employed in nearby towns, do not continue sheep husbandry, but either switch into agritourism or come to the mountains for the weekend or during the holidays.

Karst cultural landscape is characterized by fences with stone walls and fenced hay meadows. Some land is still jointly owned by members of the agricultural community, the so-called “komunele” or “gmajne” (from German die Gemainde, in the translation community) [26].

Utilised arable land and meadows in the wider area (19,890 ha) occupy 1908 ha (75.8% of utilised agricultural land, and 8.6% of the total surface), and predominantly karst pastures of low quality cover 608 ha (24.2% of utilised agricultural land) [27, 28].

Most of the pasture land is undergoing further succession to woodlands, which occupy 13,611 ha (68.4% of the total surface) [28]. These are the data for the areas of Buzet, Lanišće and Lupoglav, which are the only available data. In the narrower area of Ćićarija the share of agricultural land, especially arable land, is expected to be significantly smaller. Local people even today use a mixture of three languages, Croatian, Slovenian and Italian, easily switching between them. Additionally there is a community of Istro-Romanians who have their own language and traditions (village of Žejane), they probably arrived in Istria in around the fourteenth century from the Carpathians.

The population of the Croatian villages of the Ćićarija speak both the Čakavian dialect of Croatian, which is also spoken in many other areas of Istria and along the Croatian Adriatic sea coast (Dalmatian littoral and islands) [13] and the Kajkavian dialect (near the border with Slovenia).

The northeastern border of Ćićarija follows the road Rijeka – Trieste, while the southwestern border follows the route Lupoglav – Buzet. There are 22 villages in the Croatian part, mostly in the municipality of Lanišće, with 1722 inhabitants registered during the Census of 2011 [27]. In a broader sense, the parts of Buzet and Lupoglav municipalities also belong to Ćićarija, and in that sense the total population in the area is about 5500. Today the higher mountain areas are almost abandoned, with less than 300 people remaining as permanent residents. Most of the population lives in the south eastern part of Ćićarija, in the proximity of Rijeka. However, in the higher, central part of Ćićarija there are 14 small villages with an average population of 24 people each. Only five of these villages have more than 20 inhabitants.

The population of Ćićarija is in decline, and the current population is about a third of that from 1931 (from 8445 to 1722 inhabitants). The depopulation process was the most severe in the municipality of Lanišće, where the population density is 2.7 inhabitants per square km and the proportion of older people (age > 60) is over 40% [27, 28]. The average age in the Lanišće municipality in 2011 was 49, which is 6.7 years more than in the Istria County as whole.

During the first half of the twentieth century the majority of active population on Ćićarija were working in agriculture, forestry and charcoal production. In every larger settlement there were craftsmen to provide necessary services: tailors, carpenters, blacksmiths, barbers, masons etc. There were also families running small shops or inns [29]. After WWII, due to the slow post-war recovery, poor natural resources for agriculture and high tax burdens imposed on farmers, the structure of the economy and population started to change. In 1961 almost one third of the population in Istria (31.3%) was living from agriculture, and in 2001 the share was 2.6% [30].

At present, in most of the settlements there is no livestock and the last charcoal clamp was used at the beginning of 1970-ies. Many highland meadows, that used to be mown once a year and grazed, have become abandoned with the disappearance of livestock. Scarce pieces of fertile land are used mainly for subsistence farming, or as gardens. There is no need for traditional crafts any more, and because of the depopulation, even shops and inns do not exist in small villages. Employed persons mainly commute daily to workplaces in neighbouring towns [28, 29]. The majority of the population of the research area declared themselves as Croatians (76.5%), and the second largest group is regionally affiliated (15.2%). The rest are members of different nationalities (6.9% altogether) or non-affiliated (1.4%). However, in this study only Croatian people who were born in the study area and/or spent most of their lives there were interviewed. The interviewed people are mainly retired. All of them have farming backgrounds and were either farmers or worked in Buzet or Kopar and actively maintained gardens.

## Methods

The research was carried out following the Principles of Professional Responsibility of the American Anthropological Association and the International Society of Ethnobiology Code of Ethics (2006) [31, 32]. Data were collected using semi-structured interviews, mainly applying the free listing method, accompanied by informal walks (and talks) with selected key informants, from May to September 2015. To help elicit answers, separate questions were asked about the food use of green parts and fruits as well as medicinal plants that had been personally used by the informants. Additional notes were taken, and audio recordings were made during the interviews when possible. Participants were approached outside, during their farm work, or selected based on recommendation as the most knowledgeable people in the village.

Altogether we obtained data from 50 interviews involving 76 local informants (33 single informant interviews and 17 interviews involving two or three people). There were 37 female and 39 male informants, with a mean age of 67 (age range: 33–101, median: 67).

The interviews were carried out on the territory of three municipalities: Buzet (7 settlements: Buzet, Gornja Nugla, Hum, Kompanj, Počekaji, Roč, Sveti Martin), Lanišće (11 settlements: Brest, Brgudac, Dane, Jelovice, Klenovšćak, Lanišće, Podgaće, Prapoće, Račja Vas, Slum, Vodice) and Lupoglav (1 settlement: Lupoglav).

Plant names used follow the Plant List [33].

The division between wild and domesticated species is often blurred. Several taxa (mainly fruits and aromatic herbs) listed by the informants occur both in wild and domesticated states. We included them in the species list if we observed significant wild or feral populations of these species in the study area, as in other studies on wild foods in Croatia [34–36].

## Results

Altogether 121 species were recorded as food or medicine, used or previously used in the study area (Table 1). Thirty-one species are used exclusively as food or everyday drink, 50 species are used exclusively as medicine and the use of 40 species overlaps. The mean frequency of species mentioned per questionnaire was 30 (mean no. of exclusively medicinal species 5, exclusively food species 8, and 17 from the food-medicine spectrum).

**Table 1:** Wild and feral species traditionally used for food and medicine in the study area

Scientific name	UR	Local names	Preparation method	Medicinal use	Parts Used	Purpose	Voucher no.
*Achillea millefolium* L.	26	milefiori, milefolium, stolisnik, tausendrož	dried for tea	against amenorrhea, digestive, to strengthen the body, to improve appetite, to treat women’s menopausal symptoms, for the bladder, for the stomach	L, F	Md	40115
*Alcea rosea* L.	1	slez crveni	dried for tea	diuretic	F	Md	-
*Allium ampeloprasum* L.	11	čebula, čebüv, dibji česan, diblji česon, diblji luk, divlji luk, divlji lük, luk, poriluk diblji, purić	fried with eggs, raw as salad, raw for livestock		L, R	Fd	-
*Allium ursinum* L.	5	medvedka, medvjeđi luk	boiled, raw or dried as spice, fried with eggs, raw as salad		L	Fd	-
*Allium* sp.	4	česan, česän, divlji česan	raw, cooked		L, R	Fd	-
*Althaea officinalis* L.	10	beli slez, kapitvanjica, sirići, slez	dried for tea	to clean the sinuses, to calm coughing	F, R, L	Md	-
*Amaranthus retroflexus* L.	7	diblja blitva, divlji šćir, šćir	boiled, boiled with fennel, dried for tea, soaked in water	veterinary: against diarrhea	S, L	Md & Fd	41804
*Arctium lappa* L.	2	čičak	dried for tea, macerated	for detoxification, for better hair growth	R, FR	Md	-
*Armoracia rusticana* P. Gaertn., B. Mey. et Scherb.	1	hren	raw as salad		R	Fd	-
*Artemisia absinthium* L. ^a^	15	pelen, pelin	boiled, dried for tea, raw in liqueur	against gastritis, against pneumonia, to treat women’s problems, liver cleaning	L	Md	40142
*Arum maculatum* L.	1	kozlac	dried for tea or ointment	against haemorrhoids, to treat laryngitis and bronchitis, anti-ulcer, anti-psoriatic	L	Md	41792
*Asparagus acutifolius* L.	39	diblje šparge, šparge, šparoge, šparuge, šparüge, šporge	boiled with rice, cooked in pasta sauce, dried for tea, fried with eggs, raw or dried as spice	kidney stone relief	W	Md & Fd	-
*Bellis perennis* L.	2	margaretice, tratinčica	fried in pancakes		F	Fd	41803
*Calendula officinalis* L.	1	neven	raw in ointment	astringent, for healing the skin	F	Md	-
*Carlina acaulis* L.	2	beli trn, kravljak, vilino sito	against ageing spots on the skin, for decoration	against age spots	F, L, R	Md	-
*Carum carvi* L. ^a^	9	crni kimelj, divlji kimelj, divlji kimen, kimel, kimel divlji, kimen	dried as spice, dried for tea, raw	digestive, anti-flatulence, intestinal colic relief in children	S	Md & Fd	-
*Castanea sativa* Mill.	3	divlji kostanj, kostanj, kostanji diblji	raw	anti-rheumatic	FR	Md	-
*Centaurium erythraea* Rafn	2	kičica, taušendroža	dried for tea, raw in liqueur	digestive	W	Md	36736
*Chelidonium majus* L. ^a^	4	mlič, rosopas	dried for tea, raw- cellular juice	anti-warts, against dizziness, against skin cancer	W, L	Md	41799
*Chenopodium album* L.	29	loboda, lobodà, lübüdá	boiled, boiled with vegetables, fried, fried with eggs, raw as salad, raw for livestock, raw or dried as spice		L, W	Fd	40132, 40037
*Cichorium intybus* L.	34	cikorija, diblji radić, diblji redić, divlji radić, konjski radić, radić, vodopija, zajka, ženotrga, žutenka	boiled, boiled with vegetables, fried, as a warm drink, fried with eggs, raw as salad		R, L	Fd	40033
*Clematis vitalba* L.	20	ruj, sarabot, škrebut, tartor, trator, trtor	dried for weaving and making a kind of string for tying vineyards, fried with eggs, raw, raw for livestock, raw for weaving		L, W	Fd	40134
*Clinopodium nepeta* (L.) Kuntze (syn. *Calamintha nepetoides* Jord.)	6	dibja menta, divlja metica, metica, metvica divlja, divlji bosiljak	dried as spice, dried for tea	sedative, good for stomach	L	Md & Fd	41807
*Cornus mas* L.^a^	46	dren, drenići, drenjula, drenjule, drenjüle, drenjuli, drenjulve, drijen, drnjići, drnjole, drnjule, drnjüle, drnjuli, drünjule	cooked as compote or jam, distilled for brandy, dried for tea, fermented as vinegar, processed in wine, raw, raw in liqueur, raw in oil, raw in syrup		FR	Fd	41788-
*Corylus avellana* L.	5	divlji lešnjaki, lješnjaki	raw		S	Fd	40125
*Crataegus monogyna* Jacq.	14	beli gloh, bijeli gloh, bijeli trn, bijeli trnj, brombulje, crveni glog, crveni gloh, divlji glog, glog, glog crni, glog crveni, gloh, košići	cooked as jam, dried for tea, pressed for juice drops, raw in liqueur, raw in ointment	cardiac insufficiency treatment, blood pressure remedy	L, F, FR	Md & Fd	40113
*Daucus carota* L.	8	divlji merlin, merlin, mrkva	cooked	healing “because of carotene”	R	Md & Fd	41792
*Dioscoraea communis* (L.) Caddick & Wilkin (syn. *Tamus communis* L.)	40	blušć, blüšć, blušt, bljušć, bljušt, bljüšt	fried with eggs, raw as salad, raw for livestock, raw in liqueur, raw or dried as spice	anti-rheumatic	W, R	Md & Fd	40040
*Diplotaxis tenuifolia* (L.) DC.	19	dibja rokula, diblja rokula, divlja riga, divlja rokola, divlja rokula, riga, rikula, rohuljica, rokola, rokula, rokulja, rukola	boiled with vegetables, raw		L	Fd	40041
*Elymus repens* (L.) Gould	2	pernica, pirika	dried for tea	pain relief, to treat internal diseases	R	Md	36691
*Equisetum arvense* L.	3	poljska preslica, preslica	dried for tea, raw in bath	diuretic (urine excretor)	L	Md	-
*Euphorbia cyparissias* L.	2	mličika, mličina	dried for tea, raw	for disinfection (of water), untreated serious disease, veterinary: wound healing for sheep	L	Md	41801
*Fagus sylvatica* L.	1	bukva	boiled and mixed with sheep tallow		L	Fd	41789
*Foeniculum vulgare* Mill.	31	dibji koromač, komorač, komorač diblji, koromač, kurumač, pitomi koromač	boiled, boiled with vegetables, dried for tea, fried with eggs, raw in liqueur, raw or dried as spice	for digestion	L	Md & Fd	40032
*Fragaria vesca* L.	16	dibja jagoda, diblja jagoda, divlja jagoda, fragola, fragole, preskavčići, šumska jagoda, treskavac, triskovac	raw, marmelade		FR	Fd	36787
*Gentiana lutea* L. ssp. *symphyandra* (Murb.) Hayek	11	čemerika, encian, lincura, srčanik, šulijan	dissolved in water, raw in liqueur	anti-gout, anti-rheumatic, anti-arthritis	R	Md	40028
*Helianthus tuberosus* L.	1	čičoka	raw (ground for salad)	anti-diabetes	R	Md	-
*Helichrysum italicum* (Roth) G.Don	2	smilje	in olive oil, for skin	for the skin	F, L	Md	34732
*Humulus lupulus* L.	18	bruškandol, dibji hmelj, divlji hmelj, hmej, hmelj, hmëlj, hmilj, kmiej	boiled with rice, cooked in pasta sauce, raw or dried for soup seasoning, dried for tea, fried with eggs	sedative	L	Md & Fd	40056
*Hypericum perforatum* L.	25	gospina trava, ivanjska trava, ivanjsko cvijeće, kantariol, kantarion, rože sv. Ivana, rožice sv. Ivana, trava sv. Ivana, trava sv. Marije	dried for tea, macerated, raw in liqueur, raw in oil, raw in ointment	against haemorrhoids, anti-arthritis, to treat burns, sedative, liver cleaning, for the skin, for wounds, for veins, for stomach	F, L	Md	40140, 40141
*Ilex aquifolium* L.	1	božikovina	dried for tea to treat the flu, digestive		L	Md	-
*Iris germanica* L.	2	špada	dried for tea		R	Md	41800
*Iris illyrica* Tomm.	2	lelije, perunika	dried, raw, mixed with other feed	to strengthen the organism of lactating livestock (cows, sheep)	R	Md	36804
*Juglans regia* L. ^a^	16	orah, oreh, orih	baked in cakes, boiled, raw in liqueur	digestive, appetite stimulant, strengthening organism	L, FR	Md & Fd	40035
*Juniperus communis* L. ^a^	28	brinj, cupin, smreka, smrekva, smrekva plava, smrika, smrika crna, smrika plava, smrikva, smrikva plava, šmrikva črna, šmrikva plava	chopped raw, cooked in pasta sauce, dried for tea, raw, raw in cream, raw in liqueur, raw in oil, raw or dried as spice, bundled for use as a broom	cold remedy, digestive, anti-diarrheal, anti-rheumatic, vermifuge	FR	Md & Fd	-
*Juniperus oxycedrus* L.	4	smrikva crvena, šmrikva crvena, šmrikva krvava	raw in liqueur	digestive, astringent (for digestion)	FR	Md	-
*Lamium orvala* L.	2	mrtva kopriva	boiled		W	Fd	41798
*Laurus nobilis* L.	15	lavrika, lovor	dried for tea, raw in syrup, raw or dried as spice	cold remedy	L	Md & Fd	40036
*Lavandula angustifolia* Mill.	1	lavanda	raw in oil	calms irritated skin	L, F	Md	40039
*Linum usitattisimum* L.	1	lan	dried for tea	anti-stomach problems	S	Md	36792
*Loranthus europaeus* Jacq.	1	imela žuta	raw in liqueur	panacea	W	Md	-
*Malus domestica* Borkh.	10	jaboka, jabučići, jabuka	baked in cakes, fermented as vinegar, raw in syrup	cholesterol reduction	FR	Md & Fd	-
*Malus sylvestris* Mill. ^a^	24	dibje jaboke, diblji jabučići, diblji jabuki, divlja jabuka, divljake, divlje jaboke, divlje jabučice, divlji jabučići, lesnauke, lesnjake, lesnjauke, lisnjake, rušvići	cooked as compote, cooked as jam, fermented as vinegar, processed in fruit wine, raw as snack, raw for livestock, raw in liqueur	blood pressure and cholesterol reduction	FR	Md & Fd	40029
*Malva* cf. *neglecta* L.^b^	1	sljez	dried for tea, raw in liqueur	anti-rheumatic	R	Md	40137
*Matricaria chamomilla* L. (syn. *Chamomilla recutita* (L.) Rauschert)^a^	8	kamamila, kamilica, kamomila	dried for tea	sedative, digestive	F, L	Md	-
*Melissa officinalis* L.^a^	10	matičnjak, melisa	dried as spice, dried for tea, fried with eggs, raw in syrup	sedative	L	Md & Fd	-
*Mentha longifolia* (L.) Huds.	19	menta, metica, metvica	dried for tea, raw in syrup, raw with cheese	for digestion	L	Md & Fd	40051
*Mentha spicata* L.^c^	4	kudrava metvica, metvica	dried for tea, raw in syrup	anti-stomach problems	L	Md	40052
*Morus alba* L.	20	bijela mürva, mrva bela, mrve, murva, mürva, murva bila, mürve bele, mürvice bele	baked in cakes, cooked as jam, raw, raw in liqueur, raw in syrup	as a laxative	FR	Md & Fd	40129
*Morus nigra* L.	10	črna mürva, mrva črna, murva črna, mürve črne, mürvice črne	raw, raw for livestock		FR, L	Fd	-
*Nasturtium officinale* R. Br.	2	bijela potočarka, potočarka	dried for tea, raw	liver cleansing	L, F	Md	-
*Neottia nidus-avis* (L.) Rich.	1	vuk	dried for tea	veterinary: for sheep to remain pregnant	L, F	Md	-
*Nymphaea alba* L.	1	lokvanj	raw, on the skin	ulcer extractor	L	Md	-
*Olea europaea* L. ^a^	2	maslina	dried for tea, pressed for oil, raw	for general health	L, FR	Md	-
*Ononis spinosa* L.	5	budiš, gladeš, gladiš, gladuš, trnjić, zečji trn	dried for tea	diuretic (urine excretor)	R, FR	Md	35855
*Origanum majorana* L.	3	mažorana, mažurana	dried for tea	sedative	L	Md	-
*Origanum vulgare* L.	2	mravinac, oregano, origano	chopped raw, dried, dried for tea	sedative	L	Md	35619
*Paliurus spina-christi* Mill.	2	drača	fried in fritters		F	Fd	-
*Papaver rhoeas* L.	1	divlji mak	for colouring food		F	Fd	41797
*Parietaria officinalis* L.	3	cerkvina, crkvina, šćirica, šćirika	dried for tea	for urinary tract, kidney cleansing, for suppression of bacteria in the urine	L	Md	40136
*Physallis alkekengi* L.	1	čileanska, peruanska jagoda	raw		FR	Fd	-
*Pinus nigra* J.F.Arnold	8	bor, borić, crni bor	infused in honey, raw in liqueur, raw in syrup	anti-bronchitis, anti-cough, anti-catarrh in the throat, to treat colds and the flu	L, FR	Md	40112
*Plantago lanceolata* L.	13	bokvica, terputac, traputac, trputac, trputac uskokolisni	dried for tea, raw, raw as salad, raw in syrup	anti-cough, antiseptic, for healing wounds, for toothache, healing wounds, ulcer extractor, for the bronchi	R, L	Md	41806
*Plantago major* L.^a^	3	karegica, trputac, trputac širokolisni	raw, raw in syrup	anti-cough, antiseptic, cicatrising (for healing wounds), calms irritated skin	L	Md	41805
*Plantago media* L.	1	trputac	raw	antiseptic, for healing wounds	L	Md	40054
*Polygala nicaeensis* Risso ex Koch	1	krestušac	dried	anti-cough, for the bronchi	W	Md	39189
*Portulaca oleracea* L.	6	portulac, tušć, tušt	raw, raw as salad, raw or dried as spice		L, W	Fd	-
*Primula veris* L. ssp. *columnae* (Ten.) Lüdi	2	jaglac, jaglac divlji	dried for tea, fried in pancakes	sedative	F	Md & Fd	36761
*Prunus avium* L.	14	crna trešnja, čerešnje, črešnje, črišnje, diblje črišnje, diva čerešnja, divlje čerešnje, divlje čerešnji, divlje črešnje, divlje črišnje, divlje trešnje, trešnja	dried for tea, raw in liqueur	digestive	L, FR	Md & Fd	40030
*Prunus cerasus* L.	2	višnja	raw in liqueur	digestive	FR	Md & Fd	40038
*Prunus domestica* L.	24	amuli, amulinčići, armulini, bila čespa, kreke, kreke bijele, kreke plave, sliva, sljive bijele, šliva, šljiva, šljive bijele, švršvići	cooked as compote, cooked as jam, distilled for brandy, raw, raw in liqueur		FR	Fd	40038
*Prunus dulcis* (Mill.) D.A. Webb	7	mandule, menduli, lešniki, lešnjaki, liškanji, lišnjak,	dried for tea		S	Fd	-
*Prunus mahaleb* L.	3	rešeljka, rišeljka	dried for tea		FR	Fd	40143
*Prunus persica* L. var. *platycarpa*	4	breskve, breskvice, vinogradarska breskva, vinogradarske breskve	raw in syrup		FR	Fd	-
*Prunus spinosa* L.	31	brombulići, brumbolje, brumbulje, ciborice, crni trn, crni trnj, češpa, čišpe, črni trn, črni trnj, divlji trn, drnkalići, glog, potrnki, sliva plava, trnina, trnkice	cooked as compote, cooked as jam, dried for tea, raw, raw in liqueur, raw in syrup	cardiac insufficiency treatment,	FR, F	Md & Fd	40044
*Pteridium aquilinum* (L.) Kuhn	1	bujad, paprat	boiled		L	Fd	41794
*Pyrus amygdaliformis* Vill.	20	diblje krušviće, divlja kruška, divlje fruške, divlje fruškvići, divlje kruške, divlje kruškice, divlje krušvice, divlji krušvići, fruške, fruškići, fruškvići, früšvić, hrišvići, krüšvice divlje	cooked as compote, raw in liqueur		FR	Fd	-
*Pyrus pyraster* (L.) Burgsd.	8	dibje kršve, kruške, krüšve, krušvići	baked in cakes, cooked as compote, cooked as jam, raw in liqueur, raw in syrup		FR	Fd	-
*Quercus pubescens* Willd.	6	hrast, hrast dub, želod, želot, žir	dried for tea, raw for livestock	antidiarrhea, nutraceutical	bark, FR	Md & Fd	40117
*Robinia pseudoacacia* L.	13	akacia, bagrem, diraka, drača, kacia, kacija	fried in fritters, dried for tea, fried, raw in juice, raw in syrup	digestive, anti-flu	F	Md & Fd	40123, 40048
*Rosa canina* L.^a^	38	lužar, rožarić, ružarić, sarborić, šipak, šipok, šipók, tovarski bomboni, tovorski bomboni	cooked as jam, dried for tea, raw in syrup	anti-diarrhea	FR	Md & Fd	40126
*Rosmarinus officinalis* L.	14	rožmarin, ružmarin	boiled, dried for tea, raw or dried as spice	cold remedy, anti-dandruff, rickets treatment	L	Md & Fd	40034
*Rubus caesius* L.	43	črna jagoda, črna murga, črne maline, jagoda, kupina, kupĺna, ostruge, ribidnica, rubida, rübida, rubidnica, rübidnica	cooked as jam, dried for tea, parboiled, raw, raw in liqueur, raw in syrup	anti-diarrhea, ulcer treatment	L, FR	Md & Fd	40119
*Rubus idaeus* L.	20	crvene maline, divlje maline, frambe, frambova, frambugi, jagoda, maline, maline šumske, murga, ribidnica	raw, raw in liqueur, raw in syrup		FR	Fd	40118
*Rumex acetosa* L.	7	kiseljak, šćav, šćir, šćüav, velika zelena kiselica	boiled, dried for tea, raw, raw for livestock	anti-diarrhea	W, L	Md & Fd	40138
*Ruscus aculeatus* L.	22	kukavčići, laprinac, leprin, leprinac, leprinj, vaprin, veprin, veprina	for decoration, fried with eggs, raw as salad, raw in liqueur, raw or dried as spice, carpal tunnel massage		W, R, L	Md & Fd	-
*Ruta graveolens* L.^a^	15	ruda, rüdā, ruta	dried as spice, dried for tea, raw in liqueurs	against dizziness, menstrual problems, nerve disorders, wheezing, stomach problems, rheumatism, gout, skin diseases	L	Md & Fd	35070
*Salvia officinalis* L.	30	kuš, küš, salvia, savia	boiled in milk, caramelised, dried, raw or dried as spice, dried for tea, infused in honey, raw, raw in liqueur, raw in milk, raw in oil, raw in syrup	anti-tussive, cold remedy, depurative, appetite stimulant	L	Md & Fd	40131, 40047
*Sambucus nigra* L.^a^	42	bask, bazak, bazg, bazga, bazgovina, bezga	fried in fritters, cooked as jam, dried for tea, processed in wine, raw, raw in liqueur, raw in syrup	cicatrising	F, FR, L	Md & Fd	40128
*Sanquisorba minor* Scop.	2	dinjica, krvara	dried for tea, raw as salad	against blood clotting	L, F	Md & Fd	35949
*Satureja montana* L.	16	bresina, mačešina, mačušina, majčešina, primorski vrijesak, vrijesak, vrisak, vrisak bijeli	dried, dried as spice, dried for tea, raw as salad	for better circulation	W	Md & Fd	36782, 36781
*Satureja subspicata* Vis. (agg.)	3	majčešina, vrisak crveni	dried, dried for tea	astringent, antiseptic	W	Md	35952
*Sedum sexangulare* L.	2	majčina dušica žuta, sedum, žednjak	dried for tea, pressed for juice drops	cicatrising (for healing wounds), sedative	L	Md	-
*Sempervivum tectorum* L.^a^	16	bijeli žednik, bobojić, bobujić, čuvarkuća, čuvarkuće, kućni čuvar, netresk, pazikuća, tres	pressed for juice drops, raw in ointment	against earache, ulcer extractor, against haemorrhoids, against herpes zoster and warts	L	Md	40031
*Solidago virgaurea* L.	1	zlatnica	raw in liqueur		W	Md	-
*Sonchus oleraceus* L.	1	grandicel	boiled with vegetables		L	Fd	41796
*Sorbus aria* (L.) Crantz	7	bukovnica, marala, mirala, mukinja	raw (against diarrhea)	anti-diarrhea	FR	Md & Fd	40139
*Sorbus aucuparia* L.	3	bakovnica, jarebika, makunina	raw (against diarrhea)	anti-diarrhea	FR	Md & Fd	41802
*Sorbus domestica* L.	31	krušvići, krüšvići, lespuje, mokovina, mokvina, oskoruša, oskorušva, oškoršva, oškorušva, skorušve, skuršve, škoršva, škurše, škuršva, uškoršva	cooked as compote, cooked as jam, dried, dried for tea, raw, raw in liqueur, raw in syrup	anti-diarrhea	FR	Md & Fd	40053
*Sorbus torminalis* (L.) Crantz	3	brekuja, brekulje	dried for tea, raw	anti-diarrhea	FR	Md & Fd	-
*Symphytum officinale* L.	1	gavez	dried for tea	cicatrising (for healing wounds)	R, F, L	Md	40133
*Taraxacum* spp. sect. Ruderalia^a^	40	diblji radič, divlji radić, jajčar, konjski radič, konjski radić, maslačak, pahlenica, puhlenica, pühlenica, puhljenica, zajka, želtenica, žutenica, žutenice, žutenka, žutevka, žutevnica	boiled, boiled with rice, boiled with vegetables, cooked, cooked as jam, dried for tea, fried with eggs, infused in honey, pickled, raw as salad, raw in syrup, raw or dried as spice	against warts, depurative, kidney stone relief	W, L	Md & Fd	40135
*Teucrium chamaedrys* L.	1	dubačac, dubica	dried for tea, raw as salad	toothache remedy	L	Md	37033
*Thymus serpyllum* agg. (e.g. *Thymus longicaulis* Presl)	21	majčina dušica, timijan, timo	raw or dried as spice, dried for tea, raw as salad, raw in liqueur, raw in ointment	anti-tussive, sedative, skin treatment	L	Md & Fd	36798, 37031
*Tilia platyphyllos* Scop.	26	lipa	dried for tea, raw in syrup	sedative, cold remedy, anti-scab, irregular heartbeat remedy	L	Md	40122
*Tussilago farfara* L.	4	lepuh, podbjel, repuh	boiled for livestock, boiled with vegetables		L	Fd	41790
*Urtica dioica* L.^a^	41	kopriva, kupriva, pokriva, pukriva	boiled, boiled with vegetables, cooked with cream, dried for tea,fried with eggs, raw, raw in pancakes, raw in syrup, raw or dried as spice, soaked as fertiliser	depurative, hair treatment, anti-dandruff	L	Md & Fd	40130
*Vaccinium vitis-idaea* L.	1	brusnice			FR	Fd	-
*Verbascum thapsus* L.	4	divizma, lopuh, margaretica	dried for tea	anti-tussive, anti-flatulence, menstrual pain relief, sedative	F, L, F	Md	41795
*Viola odorata* L.	1	ljubičica	fried in pancakes		F	Fd	41793
*Viscum album* L.	8	imela, imela bijela	raw in liqueur, raw in syrup	panacea (better than *Loranthus*)	W	Md	-

Parts used: *F* flowers, *FR* fruits, *L* leaves or other green parts, *R* roots, *W* whole plants, *Fd* food use, *Md* medicinal use

^a^taxa which have also been recorded by Pieroni & Giusti (2006)

^b^Pieroni & Giusti recorded a similar species, *Malva sylvestris*

^c^Pieroni & Giusti recorded a similar species, *Mentha arvensis*

The most commonly used exclusively food species are: *Cornus mas* L., *Cichorium intybus* L., *Chenopodium album* L*., Prunus domestica* L., *Pyrus amygdaliformis* Vill*., Rubus idaeus* L., *Clematis vitalba* L., *Diplotaxis tenuifolia* (L.) DC., *Fragaria vesca* L. and *Allium ampeloprasum* L. The commonest species used exclusively as medicine are: *Achillea millefolium* L., *Tilia platyphyllos* Scop., *Hypericum perforatum* L., *Sempervivum tectorum* L., *Artemisia absinthium* L., *Plantago lanceolata* L., *Gentiana lutea* L. ssp. *symphyandra* (Murb.) Hayek, *Althaea officinalis* L., *Matricaria chamomilla* L., and *Pinus nigra* J.F.Arnold. The most commonly used food-medicine spectrum species are: *Rubus caesius* L., *Sambucus nigra* L., *Urtica dioica* L., *Dioscoraea communis* L., *Taraxacum* spp., *Asparagus acutifolius* L., *Rosa canina* L., *Foeniculum vulgare* Mill., *Prunus spinosa* L. and *Sorbus domestica* L.

Wild plants are used for food mainly in the form of preserves (jams, juice), wild vegetables served as salad, manestra soup (served with beans or dried meat) or with omelettes (*fritaja*), herbal teas or as aromatic additives to alcohol. The mixture of wild vegetables used for the soup is called *zelenjava.* Its commonest components are *Chenopodium, Cichorium, Diplotaxis, Foeniculum* and sometimes *Humulus* or *Urtica.* Medicinal plants are usually used in the form of infusions and decoctions (“herbal teas”). Such ways of administration were recorded for 52% of medicinal plant taxa.

**Fig. 6:**
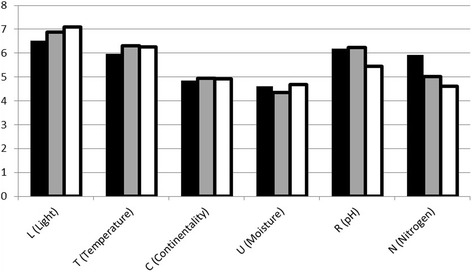
Mean Ellenberg values for exclusively food species (*black bars*), food and medicinal species (*grey bars*) and exclusively medicinal species (*white bars*)

**Fig. 7:**
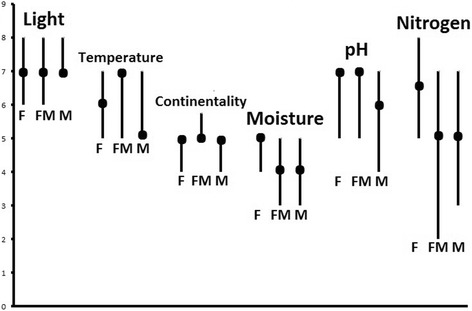
Medians (*black circles*) and first and third quartiles for Ellenberg values (values indicated by line tips). F – exclusively food species, FM – food and medicinal species, M - exclusively medicinal species

Among the most commonly used food and food-medicinal plants woody species, particularly those of woodland edges are dominant, whereas among medicinal plants it is herbaceous plants from grassland that dominate (Table 2). However, the differences between Ellenberg-Pignatti values (Figs. 3 and 4; Table 3) in the three categories of useful plants were small and statistically insignificant (Mann-Whitney U test; *P* > 0.05) apart from the difference between Nitrogen indicator value between the Exclusively Medicinal and the Exclusively Food category (*P* = 0.02). For Light and Continentality the highest indicator values were observed for medicinal plants and the lowest for food plants (Fig. 6). However median values for these two variables were identified in all three categories (Fig. 7). For Nitrogen the opposite trend was observed – the highest value (and the highest median) was observed for food plants (Figs. 6 and 7). For Temperature and Reaction (pH) the highest indicator values were observed for plants used both for food and medicine (Fig. 6).

**Table 2:** Ten most commonly used species in each of three categories

	Habitat in the studied area
Only food plants	
*Cornus mas* L.	forest fringes and clearings
*Cichorium intybus* L.	road verges, ruderal habitats
*Chenopodium album* L*.*	ruderal habitats, e.g. arable fields
*Prunus domestica* L.	forest fringes (feral)
*Pyrus amygdaliformis* Vill*.*	forest fringes
*Rubus idaeus* L.	forest firnges
*Clematis vitalba* L.	forest fringes
*Diplotaxis tenuifolia* (L.) DC.	road verges, ruderal habitats
*Fragaria vesca* L.	deciduous forests
*Allium ampeloprasum* L.	road verges, ruderal habitats
Plants from food – medicine spectrum	
*Rubus caesius* L.	forest fringes, hedges, ruderal habitats
*Sambucus nigra* L.	forest fringes, hedges, ruderal habitats
*Urtica dioica* L.	ruderal habitats
*Dioscoraea communis* L.	forest fringes, hedges
*Taraxacum* spp.	roadsides, lawns, ruderal habitats
*Asparagus acutifolius* L.	forest fringes, hedges, ruderal habitats
*Rosa canina* L.	forest fringes, hedges
*Foeniculum vulgare* Mill.	road verges, ruderal habitats
*Prunus spinosa* L.	forest fringes, hedges
*Sorbus domestica* L.	forest fringes and clearings
Purely medicinal plants	
*Achillea millefolium* L.	grasslands
*Tilia platyphyllos* Scop.	feral and cultivated in villages
*Hypericum perforatum* L.	grasslands
*Sempervivum tectorum* L.	feral in rocky places in gardens, widely cultivated
*Artemisia absinthium* L.	road verges, ruderal habitats, also cultivated
*Plantago lanceolata* L.	road verges, grasslands
*Gentiana lutea* L. ssp. *symphyandra* (Murb.) Hayek	dry grasslands
*Althaea officinalis* L.	grasslands
*Matricaria chamomilla* L.	ruderal habitats, e.g. arable fields, also cultivated
*Pinus nigra* J.F.Arnold.	forest fringes, used for afforestation of grasslands

**Table 3:** Ellenberg-Pignatti values used in the analysis. For some species and variables the values do not exist

Scientific name	Purpose	Light	Temperature	Continen-tality	Soil moisture	Reaction	Nitrogen	Salinity
*Allium ampeloprasum* L.	food	7	7	5	3	6	5	0
*Allium ursinum* L.	food	2		5	6	7	8	0
*Amaranthus retroflexus* L.	food	9	9	7	4		9	0
*Armoracia rusticana* P. Gaertn., B. Mey. et Scherb.	food	8	6	5	5		9	0
*Bellis perennis* L.	food	9	5	4			5	0
*Chenopodium album* L.	food	7	7	5	4	5	7	0
*Cichorium intybus* L.	food	9	6	5	3	8	5	0
*Clematis vitalba* L.	food	7	7	4	5	7	7	0
*Cornus mas* L.	food	6	7	6	5	8	4	0
*Corylus avellana* L.	food	6	5	4	5	5	8	0
*Diplotaxis tenuifolia* (L.) DC.	food	8	7	5	4	6	5	0
*Fagus sylvatica* L.	food	3	5	4	5		7	0
*Fragaria vesca* L.	food	6		4	4		5	0
*Lamium orvala* L.	food	3	5	5	6	7	8	0
*Morus nigra* L.	food	8	7	5	5	5	5	0
*Paliurus spina-christi* Mill.	food	7	8	6	3	7	3	0
*Papaver rhoeas* L.	food	6	6	5	5	7		0
*Physallis alkekengi* L.	food	6	7	5	7	5	6	0
*Portulaca oleracea* L.	food	7	8	5	4	7	7	0
*Prunus domestica* L.	food							
*Prunus dulcis* (Mill.) D.A. Webb	food							
*Prunus mahaleb* L.	food	7	5	6	3	8	2	0
*Prunus persica* L. var. *platycarpa*	food							
*Pteridium aquilinum* (L.) Kuhn	food	6	5	4	6	3	3	0
*Pyrus amygdaliformis* Vill.	food	7	8	4	4	7	3	0
*Pyrus pyraster* (L.) Burgsd.	food	6	5	5	6	7	7	0
*Rubus idaeus* L.	food	7	4		5	5	8	0
*Sonchus oleraceus* L.	food	7	5		4	8	8	0
*Tussilago farfara* L.	food	8		5	6	8	7	0
*Vaccinium vitis-idaea* L.	food	5	3	5	4	2	2	0
*Viola odorata* L.	food	5	6	5	5		8	0
*Achillea millefolium* L.	med.	8			4		5	0
*Alcea rosea* L.	med.	9	8	5	3	6	4	0
*Althaea officinalis* L.	med.	7	6	6	7	7	6	0
*Arctium lappa* L.	med.	9	5	5	5	7	9	0
*Artemisia absinthium* L.	med.	9	6	7	4		8	0
*Arum maculatum* L.	med.	3	6	5	7	7	8	0
*Calendula officinalis* L.	med.	8	7	5	4	5	4	0
*Carlina acaulis* L.	med.	7		4	4	0	2	0
*Castanea sativa* Mill.	med.	5	8	6		4		0
*Centaurium erythraea* Rafn	med.	8	6	5	5	6		0
*Chelidonium majus* L.	med.	6	6		5		8	0
*Elymus repens* (L.) Gould	med.	7		7	5		8	0
*Equisetum arvense* L.	med.	6			6		3	0
*Euphorbia cyparissias* L.	med.	7	7	5	3	5	5	0
*Gentiana lutea* L. ssp. *symphyandra* (Murb.) Hayek	med.	8	4	5	4	4	2	0
*Helianthus tuberosus* L.	med.	8	7	5	7		6	0
*Helichrysum italicum* (Roth) G.Don	med.	8	8	5	4	3	2	0
*Hypericum perforatum* L.	med.	7	8	6				0
*Ilex aquifolium* L.	med.	4	5	4	5	4	5	0
*Iris germanica* L.	med.	7	7	5	3	5	4	0
*Iris illyrica* Tomm.	med.							
*Juniperus oxycedrus* L.	med.	8	8	0	3	0	2	0
*Lavandula angustifolia* Mill.	med.	11	5	4	3	2	2	0
*Linum usitattisimum* L.	med.	9	7	5	4	3	3	0
*Loranthus europaeus* Jacq.	med.	7	6	6	5			0
*Malva alcea* L.	med.	8	6	4	5	8	8	0
*Matricaria chamomilla* L.	med.	7	5	5	6	5	5	0
*Mentha spicata* L.	med.	7	6	5	8	8	6	0
*Nasturtium officinale* R. Br.	med.	7	4	5	11	7	7	0
*Neottia nidus-avis* (L.) Rich.	med.	2	5	5	5	7	5	0
*Nymphaea alba* L.	med.	8		5	12	7	7	0
*Olea europaea* L.	med.	11	10	4	1		2	0
*Ononis spinosa* L.	med.	8	6	5			3	0
*Origanum majorana* L.	med.	7	7	6	4	5	3	0
*Origanum vulgare* L.	med.	7	6	5	3		3	0
*Parietaria officinalis* L.	med.	4	8	4	5	7	7	0
*Pinus nigra* J.F.Arnold	med.	7	7	4	2	9	2	0
*Plantago lanceolata* L.	med.	6	7	5				0
*Plantago major* L.	med.	8			5		7	0
*Plantago media* L.	med.	7		7	4	8	3	0
*Polygala nicaeensis* Risso ex Koch	med.	8	6	5	3	7	2	0
*Satureja subspicata* Vis.	med.	8	5	7	4	7	3	0
*Sedum sexangulare* L.	med.	7	5	4	7	8	1	0
*Sempervivum tectorum* L.	med.	8	5	5	2	4		0
*Solidago virgaurea* L.	med.	5			5		5	0
*Symphytum officinale* L.	med.	7	6	4	8		8	0
*Teucrium chamaedrys* L.	med.	7	6	5	2	8	1	0
*Tilia platyphyllos* Scop.	med.	3	5	4	5		7	0
*Verbascum thapsus* L.	med.	8		4	4	7	7	0
*Viscum album* L.	med.	7	5	5	0	0	0	0
*Amaranthus retroflexus* L.	med. & food	9	9	7	4		9	0
*Asparagus acutifolius* L.	med. & food	6	9	4	2	5	5	0
*Carum carvi* L.	med. & food	8	4	5	5		6	0
*Clinopodium nepeta* (L.) Kuntze (syn. *Calamintha nepetoides* Jord.)	med. & food	5	7	5	3	9	3	0
*Crataegus monogyna* Jacq.	med. & food	6	7	5	4	6	3	0
*Daucus carota* L.	med. & food	8	6	5	4	5	4	0
*Dioscoraea communis* (L.) Caddick & Wilkin (syn. *Tamus communis* L.)	med. & food	5	7	5	5	8	6	0
*Foeniculum vulgare* Mill.	med. & food	9	8	5	3	7	7	0
*Humulus lupulus* L.	med. & food	7	6	4	8	6	8	0
*Juglans regia* L.	med. & food	6	6	6	5	6	6	0
*Juniperus communis* L.	med. & food	8	0	0	4	0	4	0
*Laurus nobilis* L.	med. & food	2	7	4	8	4	6	0
*Malus domestica* Borkh.	med. & food	7	7	5	5	5	5	0
*Malus sylvestris* Mill.	med. & food	8	6	4	4		8	0
*Melissa officinalis* L.	med. & food	6	7	5	4	6	4	0
*Mentha longifolia* (L.) Huds.	med. & food	7	5	5	8	8	8	0
*Morus alba* L.	med. & food	8	7	5	5	5	5	0
*Primula veris* L. ssp. *columnae* (Ten.) Lüdi	med. & food	7		3	4	8	3	0
*Prunus avium* L.	med. & food	4	5	6	5	7	5	0
*Prunus cerasus* L.	med. & food	9	7	6	5	5	5	0
*Prunus spinosa* L.	med. & food	7	5	5				0
*Quercus pubescens* Willd.	med. & food	7	8	6	3	7	4	0
*Robinia pseudoacacia* L.	med. & food	5	7	5	4		8	0
*Rosa canina* L.	med. & food	8	5	5	4			0
*Rosmarinus officinalis* L.	med. & food	11	8	4	2	6	1	0
*Rubus caesius* L.	med. & food	7	5	5	7	7	9	0
*Rumex acetosa* L.	med. & food	4	8	5	4	5	5	0
*Ruscus aculeatus* L.	med. & food	8				4	5	0
*Ruta graveolens* L.	med. & food	9	7	6	3	7	2	0
*Salvia officinalis* L.	med. & food	11	6	6	2	7	1	0
*Sambucus nigra* L.	med. & food	7	5	4	5		9	0
*Sanquisorba minor* Scop.	med. & food	7	6	5	3	8	2	0
*Satureja montana* L.	med. & food	8	6	6	3	7	2	0
*Sorbus aria* (L.) Crantz	med. & food	6	5	5	4	7	3	0
*Sorbus aucuparia* L.	med. & food	6						0
*Sorbus domestica* L.	med. & food	4	7	5	3	8	3	0
*Sorbus torminalis* (L.) Crantz	med. & food	4	6	5	4	7	4	0
*Taraxacum* spp. sect. Ruderalia	med. & food	7			5		7	0
*Thymus serpyllum* agg. (e.g. *Thymus longicaulis* Presl)	med. & food	7	7	7	4	7	3	0
*Urtica dioica* L.	med. & food				6		8	0

## Discussion

The small differences between Ellenberg values for medicinal and food plants can probably be explained by the fact that both these groups incorporate species with very diverse organs used and very diverse phytochemical composition. When designing this study we did however expect that the Light indicator values for medicinal plants would be higher than those of food plants, as many of the medicinal plants grow on dry pastures and roadsides and contain aromatic essential oils. Such a difference actually does occur in our data, but it is not significant. Dry-habitat plants from the Lamiaceae family (13 taxa in our study), rich in essential oils, are some of the basic elements of both local traditional pharmacopeias in the Mediterranean and in Central Europe, and the medicinal plants recommended by written official pharmacopeias. They are particularly useful in healing digestive problems and fighting microbial infection. However, these are usually a few species in a given set of locally used species, and their effect was not significant in a larger matrix of data with species containing different kinds of medicinally active substances. The significant difference in Nitrogen value probably arose due to the fact that several edible plants are nitrophilous weeds, whereas among medicinal plants, as previously mentioned, there are many dry grassland species growing on skeletal soils.

In the use of food the total caloric value is very important and it can be better achieved with higher biomass yields, which are obviously more likely in nitrogen-rich habitats. Most edible green vegetables are plants growing in cultivated crops, on field edges, road margins and other locations in the home yards which have been either intentionally manured, often for centuries, or unintentionally fertilized with human and animal excrements and urine. As a larger biomass of is needed, for food plants compared to medicinal plants, they are more likely to be gathered in a closer vicinity to the house than in these fertilized agro-ecosystems.

The species used in the area are a mix of species typically used in the Mediterranean and in Central Europe, which reflects the character of the vegetation in the study area, intermediate between the two zones. All the species used were reported in other ethnobotanical works from Croatia or neighbouring countries, e.g. [34–44]. A particular feature of the local cuisine is scrambled eggs (*fritaja*) prepared with young shoots of *Ruscus aculeatus* and *Cannabis sativa* seeds and oil, used against diabetes and high pressure, and to boost immunity. It is interesting that *Humulus lupulus* is still used as a vegetable, unlike on the north Adriatic island of Krk, where people used to use it, but it is now completely forgotten [36]. Previous studies also paid attention to a relatively “unoriginal” list of plant remedies used in Ćićarija [13, 17]. However, this may be explained by the fact that phytotherapy was quite developed in this part of Europe, hence most medicinal plants in the area had already been described in detail in Renaissance herbals. A similar high correspondence between the written pharmacopeias and the present choice of plants used can be found in other European countries as well [45–47]. Compared to the previous study of Croatian plant folk remedies in Ćićarija [13] we found more plant remedies used (even when the list was restricted to the same villages studied by Pieroni & Giusti). Pieroni and Giusti mentioned 25 plants. We confirmed the use of all the wild and semi-wild species mentioned by them (19 taxa), except for two taxa where we found the use of a different species but within the genus mentioned in their work (*Malva* and *Mentha*). In our study we found 54 species of plants mentioned by our respondents in the villages studied by those authors (Jelovice, Dane, Vodice, Trstenik, Račja Vas, Lanišće, Prapoće, Brgudac). It is difficult to compare these studies as in this previous work the number of respondents and frequency of citation was not mentioned. We can however hypothesize that the larger number of species recorded in our study stems mainly from two factors. First of all the previous researchers were outsiders speaking Italian to local residents, who may not have been able to express everything in Italian. The first author of our study has spent twelve years researching Ćićarija and thus knows many people well, which may have given respondents more confidence. Secondly, in recent years more and more literature on herbal remedies has become available to the general public, which may result in some new uses, the re-invention of old uses or help in the remembrance of old uses [45, 46]. This may have happened even in spite of our intention not to record “literature” uses little ingrained in the habits of local people, or very recently acquired.

## Conclusions

The differences between Ellenberg values of medicinal and food plants collected in the study area are negligible. The only significant differences were detected for the Nitrogen value. This is probably caused by the fact that edible green vegetables are mainly species of nitrogen-rich agro-ecosystems. It could be interesting to carry out a similar study in some other regions of Europe where Ellenberg values are accessible in order to see if the results we achieved show a typical pattern in the relation between food and medicinal plants.
